# Social Behavior and Ultrasonic Vocalizations in a Genetic Rat Model Haploinsufficient for the Cross-Disorder Risk Gene *Cacna1c*

**DOI:** 10.3390/brainsci11060724

**Published:** 2021-05-29

**Authors:** Markus Wöhr, Theresa M. Kisko, Rainer K.W. Schwarting

**Affiliations:** 1 Social and Affective Neuroscience Research Group, Laboratory of Biological Psychology, Research Unit Brain and Cognition, Faculty of Psychology and Educational Sciences, KU Leuven, B-3000 Leuven, Belgium; 2 Leuven Brain Institute, KU Leuven, B-3000 Leuven, Belgium; 3 Faculty of Psychology, Experimental and Biological Psychology, Behavioral Neuroscience, Philipps-University of Marburg, D-35032 Marburg, Germany; kiskot@staff.uni-marburg.de (T.M.K.); schwarti@staff.uni-marburg.de (R.K.W.S.); 4 Center for Mind, Brain, and Behavior, Philipps-University of Marburg, D-35032 Marburg, Germany

**Keywords:** Cav1.2, calcium, animal model, rough-and-tumble play, social play, social approach, ultrasonic vocalization, playback, social contact call, alarm call

## Abstract

The top-ranked cross-disorder risk gene *CACNA1C* is strongly associated with multiple neuropsychiatric dysfunctions. In a recent series of studies, we applied a genomically informed approach and contributed extensively to the behavioral characterization of a genetic rat model haploinsufficient for the cross-disorder risk gene *Cacna1c.* Because deficits in processing social signals are associated with reduced social functioning as commonly seen in neuropsychiatric disorders, we focused on socio-affective communication through 22-kHz and 50-kHz ultrasonic vocalizations (USV). Specifically, we applied a reciprocal approach for studying socio-affective communication in sender and receiver by including rough-and-tumble play and playback of 22-kHz and 50-kHz USV. Here, we review the findings obtained in this recent series of studies and link them to the key features of 50-kHz USV emission during rough-and-tumble play and social approach behavior evoked by playback of 22-kHz and 50-kHz USV. We conclude that *Cacna1c* haploinsufficiency in rats leads to robust deficits in socio-affective communication through 22-kHz and 50-kHz USV and associated alterations in social behavior, such as rough-and-tumble play behavior.

## 1. Introduction

The top-ranked cross-disorder risk gene *CACNA1C* is strongly associated with multiple neuropsychiatric dysfunctions. This includes affective disorders, namely depression [1] and bipolar disorder [2], as well as neurodevelopmental disorders, most notably autism spectrum disorder [3] and schizophrenia [4]. *CACNA1C* encodes the pore-forming α1C subunit of the voltage-gated L-type calcium channel (LTCC) Cav1.2. Characterized by a broad tissue expression profile with high expression levels in the central nervous system, Cav1.2 accounts for the majority of all LTCCs in the brain. It is strongly involved in the regulation of depolarization-dependent calcium influx into the cell, triggering intracellular signaling cascades including major pathways involved in neuronal plasticity processes [5].

Single-nucleotide polymorphisms (SNPs) within *CACNA1C* are amongst the best replicated and most robust genetic findings from genome-wide association studies (GWAS) in psychiatry [6]. Up to now, the exact molecular consequences of *CACNA1C* SNPs associated with neuropsychiatric dysfunctions are not fully understood. Often, such SNPs are intronic and it is widely believed that they exert their effects through altering *CACNA1C* gene expression. Albeit not consistently, a substantial number of studies reported such SNPs to result in decreased expression levels [7,8,9,10,11,12]. For instance, CACNA1C expression levels were found to be decreased in postmortem analyses of brain samples from risk SNP carriers in bipolar disorder [9] and schizophrenia [10], possibly because of inhibitory transcriptional regulation through chromosomal looping [11]. A better understanding of the functional effects of *CACNA1C* dosage and how reductions in *CACNA1C* gene expression alter behavioral phenotypes with relevance to neuropsychiatric dysfunctions is therefore needed.

In a recent series of studies, we applied a genomically informed approach and contributed extensively to the behavioral characterization of a genetic rat model haploinsufficient for the cross-disorder risk gene *Cacna1c* [13,14,15,16,17,18,19], flanked by neurobiological analyses [20,21,22,23]. In this rat model, Cav1.2 expression in the brain is reduced to about 50% of wildtype littermate controls, both in males [15] and females [16]. In a significant subset of our studies, we focused on social behavior and ultrasonic vocalizations because reduced social functioning was associated with *CACNA1C* SNPs and is commonly seen in neuropsychiatric disorders in humans [15,16,17,18,19]. For example, *CACNA1C* SNPs were associated with reduced socio-affective information processing capacities. This was reflected in altered facial emotion recognition and social outgroup processing [24,25,26]. Verbal fluency was reported to be reduced in *CACNA1C* SNP risk carriers [27]. 

## 2. Social Behavior in Rats

Wild rats live in large social colonies with overlapping generations, often in underground burrow systems with shared tunnels and chambers. Such colonies are typically structured into subgroups, with prominent near-linear dominance hierarchies, particularly in males. In line with the complexity of their social environment, the social life of rats is characterized by a broad variety of social behaviors [28]. 

In adult rats, the social repertoire includes reproductive behaviors, with both sexes being highly promiscuous. In fact, there is little evidence for mate choice and stable pair bonds. Strong bonds, however, are formed between mother and their infants. Maternal caregiving behavior ranges from nursing to licking and grooming. Another important feature of the social repertoire displayed by adult rats are agonistic behaviors, usually directed against intruders from outside the colony. Males were found to be less socially tolerant than females, with males but not females patrolling and defending territory boarders. In females, aggressive behavior was found to be low except during lactation. Other prominent aspects of the social life of rats are huddling and social grooming [29]. 

In juvenile rats, a very prominent component of their social repertoire is play fighting, also called rough-and-tumble play [30]. As the first social behavior not directed towards the mother, it is widely believed that rough-and-tumble play helps to prepare the young rats to develop important social skills needed in a complex social environment. In fact, rough-and-tumble play does not only contain sexual and aggressive behavioral components, but it also requires the fast integration of multiple sources of social information during the selection of appropriate behavioral responses. Rough-and-tumble play is initiated by one rat approaching another one and attempting to touch its neck with the snout, called nape contact or pouncing. During rough-and-tumble play, rats wrestle, box, and kick each other with the aim to turn the play partner on the back and to pin it. Regular attempts to escape lead to chasing behavior. A major difference to aggressive interactions in adult rats is that juvenile rats take turns. Rough-and-tumble play thus lacks a clear dominance pattern with one rat dominating the other rat. Typically, males engage in rough-and-tumble play more than females [31]. Rough-and-tumble play is highly rewarding to juvenile rats [32]. 

While it is notoriously difficult to observe rats in the wild due to the fact that they are nocturnal and have a sub-terrestrial lifestyle, available evidence suggests that laboratory rats display the full behavioral repertoire of wild rats, albeit wild rats were reported to be more aggressive and less playful [28]. Moreover, in line with the daily demands posed by living in a complex social environment, laboratory studies showed that rats can differentiate between individuals. The ability to recognize others is not only relevant for discriminating colony members from intrudes but also within the colony, for instance in the context of mate and food competition [33]. Consistent with studies on social transmission of food preferences in wild rats, laboratory studies demonstrated that rats can learn about positive and negative events through observing others. This includes the transfer of information concerning distant foods [34] and observational learning of fear [35]. Laboratory studies further revealed that rats engage in cooperative actions [36], prefer mutually rewarding options [37], follow rules of general and direct reciprocity [38], and display empathy-driven helping behavior [39].

## 3. Ultrasonic Vocalizations in Rats

**Figure 1:**
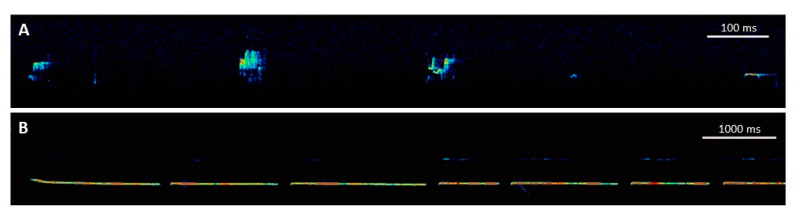
Exemplary spectrograms of ultrasonic vocalizations (USV) emitted by rats. (**A**) Spectrogram of 50-kHz USV serving as social contact calls. Please note the presence of different 50-kHz USV subtypes, including FLAT, STEP, TRILL, and MIXED calls. (**B**) Spectrogram of 22-kHz USV serving as alarm calls. Please note the difference in time scaling.

The rich social repertoire of rats includes the emission of vocal signals, so called ultrasonic vocalizations (USV) [40,41]. As opposed to sonic vocalizations, they are not audible to humans because their sound frequency is too high for the human hearing system. Typically, three main types are distinguished, fulfilling multiple biological functions as socio-affective signals [42]. The first type that occurs during early ontogeny are 40-kHz USV emitted by pups in response to separation from mother and littermates within the first two weeks of life [43]. They play a fundamental role in maintaining close mother–pup interactions and 40-kHz USV were shown to induce search and retrieval behavior in the mother, particularly during lactation [44]. Later, in juvenile and adult rats, two different types are prevalent, with their occurrence being strongly dependent on the emotional valence of the situation (Figure 1). In situations characterized by a negative valence, such as predator exposure, 22-kHz USV occur [45]. Such 22-kHz USV induce behavioral inhibition in recipients, suggesting that they serve as alarm calls [46]. In contrast, 50-kHz USV occur in situations characterized by a positive valence, typically appetitive social interactions, such as rough-and-tumble play [47] and mating [48] and were dubbed “rat laughter” [49]. Such 50-kHz USV are characterized by a high level of complexity and many different 50-kHz USV subtypes were described. However, no consensus has been reached on how many different 50-kHz USV subtypes exist and recently applied classification systems, for instance, differentiate between two [50], four [51], or even fourteen [52] subtypes. Commonly distinguished 50-kHz USV subtypes include FLAT, STEP, TRILL, and MIXED calls. It is believed that the frequency-modulated subtypes of 50-kHz USV, i.e., STEP, TRILL, and MIXED calls, are most closely associated with positive affect and reward. There is strong evidence indicating that 50-kHz USV fulfill affiliative functions [53]. 

### 3.1. Rough-and-Tumble Play and Ultrasonic Vocalizations in Rats

Juvenile rats emit 50-kHz USV in anticipation and during rough-and-tumble play [47]. Anticipatory 50-kHz USV were found to occur at high levels in a context associated with rough-and-tumble play and to increase over days with repeated play sessions. This suggests that the emission of 50-kHz USV is driven by the anticipation of a rewarding social interaction and that it reflects wanting [54]. In fact, while rats without prior play experience vocalized very little during the exposure to a conspecific separated by a screen, high rates of 50-kHz USV occurred under such conditions after a single opportunity to play and rats emitted even more 50-kHz USV than during rough-and-tumble play. It was recently suggested that the reduced emission of anticipatory 50-kHz USV could reflect a depression-related behavioral phenotype [55]. 

During rough-and-tumble play, the highest numbers of 50-kHz USV occur during actual play phases as opposed to non-playful social interactions [15]. Moreover, it was shown that the emission of 50-kHz USV changes as a function of rough-and-tumble play behavior [56] and 50-kHz USV emission was found to be correlated across individuals with appetitive components of the rough-and-tumble play repertoire, such as dorsal contacts [47]. Later, more detailed spectrographic analyses were performed and revealed that this correlation was essentially driven by the frequency-modulated subtypes of 50-kHz USV, i.e., STEP, TRILL, and MIXED calls, primarily associated with liking [56]. Together, this indicates that 50-kHz USV reflect wanting or liking, depending on the time point of emission [54]. 

Synchronized recordings with high temporal resolution further allowed insights into how 50-kHz USV are integrated into the rough-and-tumble play repertoire [15]. The synchronized analyses revealed that particularly high 50-kHz USV rates occur during wrestling and chasing. Relatively low numbers are emitted during pinning, with 50-kHz USV emission rates similar to non-play phases. Considering the fast-paced actions displayed during rough-and-tumble play, this indicates that there is a fine temporal control of 50-kHz USV emission and that 50-kHz USV emission and specific components of the rough-and-tumble play repertoire are precisely coordinated. However, not all 50-kHz USV subtypes occur at similar rates. The prevalence is highest for FLAT, TRILL, and MIXED calls, while STEP calls occur les often. This holds true for pinning, wrestling, and chasing, suggesting that distinct 50-kHz USV subtypes are not associated with specific components of the rough-and-tumble play repertoire within individuals [15]. Other studies support a precise temporal control. By focusing on a limited time window before and after a playful interaction, it was shown that 50-kHz USV occur at particularly high rates immediately before a playful attack and that different 50-kHz USV subtypes are associated with specific components of the rough-and-tumble play repertoire, such as a short subtype, which was seen at high rates after evasions but not complete rotations [57]. 

In line with the more prominent rough-and-tumble play behavior in males [31], emission of 50-kHz USV was repeatedly found to be higher in males than females [16,57]. In particular, FLAT and STEP calls are higher in males than in females. Overlapping calls also occur in higher rates in males than females [17]. The peak frequency of 50-kHz USV emitted by males was found to be lower than in females [17]. Likewise, the close association between 50-kHz USV emission and specific components of the rough-and-tumble play repertoire was shown to be most prominent in males, while not clearly present in females [17]. Moreover, there is also evidence for strain differences. During rough-and-tumble play, more 50-kHz USV were found to be emitted by Sprague–Dawley than Wistar rats, with Wistar rats engaging less in rough-and-tumble play behavior [58,59]. 

The high prevalence of 50-kHz USV in anticipation and during rough-and-tumble play and their fine temporal integration into the rough-and-tumble play repertoire suggest that 50-kHz USV promote and maintain playful social interactions by functioning as play signals and/or social contact calls. This view is supported by deafening experiments and devocalization studies. For instance, pinning was found to be diminished in deafened rats, while play initiation through dorsal contacts remained unchanged [60]. Moreover, a series of experiments with rats that were unable to vocalize due to surgical devocalization demonstrated that lack of socio-affective communication through 50-kHz USV leads to a disruption of rough-and-tumble play behavior [61,62,63]. In pairs of devocalized rats, rough-and-tumble play behavior was clearly reduced. Devocalized rats launched fewer playful attacks, displayed an increase in startle responses when contacted by the play partner, and were more likely to defend themselves in response to a playful attack [62]. Moreover, a related study found that rough-and-tumble play behavior is lower in intact pairs that were housed with devocalized cage mates than in intact pairs housed with intact cage mates. This suggests that rough-and-tumble play helps to learn about the social functions of 50-kHz USV. An intact rat exclusively engaging in playful interactions with a devocalized cage mate might lack necessary input associated with hearing 50-kHz USV during the critical time window of the rough-and-tumble play period [63]. 

In dyads with one devocalized and one intact rat, the effects were milder but alterations in specific components of the rough-and-tumble play repertoire, such as defensive responses, were repeatedly observed [61,62]. Moreover, evidence was provided in support of the notion that the rat that is pinning is emitting 50-kHz USV because 50-kHz USV emission was clearly higher when the intact rat was pinning than when it was pinned by the devocalized partner [63]. Other aspects of the rough-and-tumble play repertoire, however, were not affected by surgical devocalization. As reported before [57], 50-kHz USV were more frequent before playful contact is made than when such contact is terminated, and this pattern remained intact despite surgical devocalization of one of the play partners [62]. Interestingly, pre-contact 50-kHz USV were just as frequent as those of the devocalized play partner initiated the playful interaction as when an intact one was. This suggests that 50-kHz USV are not only emitted by the initiator to signal a playful interaction, but also by the receiving partner, presumably because 50-kHz USV function as enticements by one rat to solicit a playful attack from another [62]. However, this view is challenged by results obtained in a preference test, where rats did not prefer to engage in rough-and-tumble play with an intact play partner over a devocalized play partner. Mute rats were found to be as attractive as rats that were able to vocalize when both were simultaneously available [62]. In fact, it was found that more playful attacks are launched against devocalized than intact play partners [61]. Together, the devocalization studies indicate that 50-kHz USV facilitate rough-and-tumble play, presumably through promoting positive affect in play partners or by the induction of social proximity.

There is compelling evidence indicating that the emission of 50-kHz USV during rough-and-tumble play is under genetic control. A prominent role of genetic factors is highlighted by three independent selective breeding studies targeting distinct behavioral domains. In a first selective breeding study, rats were selected depending on their tendencies to emit low versus high rates of 50-kHz USV in response to rough-and-tumble play mimicked by a human experimenter through tickling [64,65]. Within a few generations, prominent line differences in the emission of 50-kHz USV were detected and rough-and-tumble play was found to be altered. Already in the fourth generation, the emission of 50-kHz USV during rough-and-tumble play was highest in the high line. In the low line, 50-kHz USV were virtually absent. An unselected random control line emitted moderate numbers of 50-kHz USV [65]. Consistent with the idea that 50-kHz USV facilitate rough-and-tumble play, changes in the emission of 50-kHz USV were associated with alterations in rough-and-tumble play and pinning was found to be clearly affected. Mirroring the 50-kHz USV emission pattern, pinning was highest in the high line and lowest in the low line, with the random line displaying an intermediate phenotype [65]. Of note, similar findings were obtained in a replication of the selective breeding study using more detailed spectrographic analyses of 50-kHz USV emission [66,67,68] and linked to an autism-related behavioral phenotype [69]. 

A second selective breeding study assessed 50-kHz USV emission during rough-and-tumble play in rats selected for low versus high levels of separation-induced 40-kHz USV as pups [70]. It was found that in both lines rough-and-tumble play behavior and 50-kHz USV emission were lower than in a random control line. This indicates that low social motivation as seen in the low line is associated with reduced 50-kHz USV emission. However, this also indicates that anxiety plays a modulatory role. Rats selected for high rates of separation-induced 40-kHz USV as pups are characterized by high trait anxiety and display enhanced anxiety-related behavior on the elevated plus maze in adulthood [71]. This suggests that high trait anxiety is associated with lower 50-kHz USV emission during rough-and-tumble play [72]. 

In fact, in a third selective breeding study, rats were selected for low versus high anxiety-related behavior on the elevated plus maze and through this means it was confirmed that 50-kHz USV emission is strongly affected by trait anxiety [73]. Rats selectively bred for high anxiety levels engaged less in rough-and-tumble play and emitted fewer 50-kHz USV than rats selectively bred for low anxiety levels or a random control line. The effects of selective breeding were strong and 50-kHz USV emission was almost completely absent during rough-and-tumble play in rats characterized by high trait anxiety [73]. Together, the selective breeding studies [64,65,66,67,68,69,70,71,72,73] demonstrate that the emission of 50-kHz USV is a heritable trait, for which rats can be selected and that is negatively associated with trait anxiety.

Besides genetic factors, the environment has a strong impact on the emission of 50-kHz USV. For instance, play deprivation for about three weeks through individual housing was found to enhance 50-kHz USV emission during rough-and-tumble play, while aversive stimuli, such as bright white light reduced 50-kHz USV emission [47]. Other social experiences, such as social rejection, might also have a negative impact [74]. 

Various psychoactive agents affect the emission of 50-kHz USV during rough-and-tumble play. This includes morphine [58], amphetamine [58], and the endocannabinoid signaling modulator URB597 [59]. However, their effects were often complex and dependent on other factors, such as strain. For instance, morphine enhanced 50-kHz USV emission during rough-and-tumble play in Sprague–Dawley rats but not Wistar rats and this effect was most prominent during the initial playful encounters [58]. In contrast, amphetamine inhibited 50-kHz USV emission during rough-and-tumble play but enhanced 50-kHz USV during non-social activities, such as cage exploration and self-grooming, in Sprague–Dawley rats but not Wistar rats [58]. Moreover, the anandamide hydrolysis inhibitor URB597 was reported to enhance 50-kHz USV emission during rough-and-tumble play depending on strain and the aversiveness of the situation [59]. 

A prominent role in modulating the emission of 50-kHz USV is played by the endogenous vasopressin system. While the intracerebroventricular administration of synthetic vasopressin had no effect, blocking the central vasopressin system through injections of a vasopressin 1a receptor antagonist into the brain led to a reduction in rough-and-tumble play and 50-kHz USV [73]. Other studies targeted the glutamatergic system [50,75,76] or the insulin-like growth factor I [77]. Regional brain cholecystokinin levels were found to change as a function of rough-and-tumble play behavior and thus presumably proportional to 50-kHz USV emission [78]. Although ethanol was reported to enhance social behavior, it had no effect on 50-kHz USV during rough-and-tumble play [79].

There is also a significant number of studies assessing the long-term effects of various early life stressors on 50-kHz USV emission during rough-and-tumble play. Such studies focused, for instance, on the effects of prenatal exposure to ethanol [80,81] and valproic acid [82,83]. Recent studies found that prenatal ethanol exposure leads to an enhanced emission of 22-kHz USV at the expense of 50-kHz USV during rough-and-tumble play in males but not females [84]. Likewise, it was shown that rats exposed to the viral mimic poly I:C during prenatal development engage less in rough-and-tumble play and emit fewer 50-kHz USV. Interestingly, these effects were most prominent in males [85,86]. Similar findings were obtained in a study on early life stress, including maternal separation and lipopolysaccharide injections. Early life stress was found to be associated with reduced emission of 50-kHz USV during rough-and-tumble play, an effect that could be prevented by a variety of sensory interventions during neonatal development [87]. Together, this suggests that reduced levels of 50-kHz USV during rough-and-tumble play in the sender reflect atypical development, which is relevant for a wide range of neurodevelopmental disorders in humans, such as autism and schizophrenia. 

### 3.2. Playback of Ultrasonic Vocalizations in Rats 

To study the socio-affective communicative functions of the different types of USV in rats, we developed a playback paradigm [88]. Through this means, we showed that 40-kHz USV emitted by isolated pups, but not an artificial 40-kHz sine wave tone, lead to maternal search behavior in the dam, consistent with the notion that pup 40-kHz USV help to maintain a close interaction between pup and dam [89]. We further demonstrated for the first time that 50-kHz USV typically emitted by juvenile and adult rats in appetitive social interactions, such as rough-and-tumble play, evoke social approach behavior, indicating that 50-kHz USV serve as social contact calls [88]. The social approach response is strong and is associated with a prominent increase in locomotor activity. It typically occurs within a few seconds after the playback presentation started. Often, social approach behavior during 50-kHz USV playback is followed by search behavior after the playback presentation ended. Response calls might also occur [88]. 

Importantly, strong social approach behavior is evoked exclusively in response to the playback of natural 50-kHz USV. Firstly, natural 22-kHz USV lead to behavioral inhibition in line with an alarming function [88], irrespective of the threatening stimulus that caused 22-kHz USV emission [46]. Secondly, background noise and time- and amplitude-matched white noise do not elicit social approach behavior [90]. Thirdly, albeit time- and amplitude-matched 50-kHz USV sine-wave tones do lead to social approach behavior, the response is weaker than during playback of natural 50-kHz USV [88]. 

Social approach behavior evoked by playback of 50-kHz USV is most prominent in juvenile rats and weaker in adult rats [88]. In adult rats, the social approach response is particularly strong in females [91]. Moreover, rats that emit particularly high levels of 50-kHz USV were found to display stronger social approach behavior than rats that produce few 50-kHz USV [92]. Together, this indicates that developmental aspects, sex-related factors, and personality traits related to sociability play an important modulatory role. It further suggests that rats with a higher level of social motivation, as seen for instance in juvenile rats during the rough-and-tumble play period, display stronger social approach behavior in response to 50-kHz USV playback [88]. This idea is further supported by the fact that social approach behavior was enhanced following a brief period of social isolation of 24 h [93]. 

Long-term social isolation for four weeks, however, exerted an inhibitory effect and blocked social approach behavior in response to 50-kHz USV playback in juvenile but not adult rats, suggesting that social experiences during the rough-and-tumble play period are important for rats to develop their social behavioral repertoire [93]. Corroborating evidence was obtained in rats exposed to different forms of environmental enrichment [94]. Specifically, long-term exposure to physical environmental enrichment was associated with reduced social approach behavior in response to 50-kHz USV playback. In contrast, social environmental enrichment led to enhanced social approach behavior. In fact, the reduction in social approach behavior following physical environmental enrichment was reverted by additional exposure to social environmental enrichment. 

Another important feature of the social approach response is the habituation phenomenon. Strong social approach behavior is typically only seen during the first exposure to playback of 50-kHz USV [90]. Even after a delay of one week, no prominent social approach response is seen during a second exposure. The habituation phenomenon, however, appears to be strain-dependent [95] and can be blocked by administering the amnesia-inducing drug scopolamine immediately following the first exposure [90].

At the neurobiological level, the social approach response is associated with increased neuronal activity in the nucleus accumbens, a key brain area involved in reward processing [96]. Another brain area with increased neuronal activity in response to playback of 50-kHz USV is the prefrontal cortex. While 50-kHz USV activate prefrontal cortex and nucleus accumbens, 22-kHz USV lead to an activation of the perirhinal cortex, the amygdala, and the central gray [96]. In the nucleus accumbens, 50-kHz USV playback leads to phasic release of dopamine and the strength of the dopamine response is positively associated with social approach behavior [97]. Repeated 50-kHz USV playback leads to diminished dopamine release, suggesting that the habituation phenomenon does not only occur at the behavioral but also the neurobiological level. Importantly, dopamine release is seen exclusively in response to 50-kHz USV but not background noise and time- and amplitude-matched white noise, or 22-kHz USV.

The social approach response evoked by playback of 50-kHz USV can be modulated pharmacologically. In line with the observation that increased dopamine release is associated with particularly strong social approach behavior [97], amphetamine treatment was found to result in enhanced social approach behavior [98]. The enhancing effect of amphetamine was most prominent in rats that are characterized by personality traits related to low sociability and that produce relatively few 50-kHz USV [92]. In fact, amphetamine treatment was found to boost social approach behavior in these rats in such a manner that their response was indistinguishable from the one displayed by rats emitting high rates of 50-kHz USV. A prominent modulatory role is also exerted by the opioid system [99]. Specifically, administration of the μ-opioid-receptor antagonist naloxone reduced social approach behavior in response to 50-kHz USV playback, while the μ-opioid-receptor agonist morphine enhanced the social approach response. 

Finally, we applied the 50-kHz USV playback paradigm repeatedly in rat models for neuropsychiatric dysfunctions, most notably rat models for neurodevelopmental disorders, such as autism. In a first study, we studied social behavior and ultrasonic communication in a *Shank3* deficient rat model for autism [100]. In humans, *SHANK3* mutations lead to Phelan-McDermid syndrome and are one of the most penetrant causes of autism [101]. During direct reciprocal social interactions, male but not female *Shank3* deficient rats displayed deficits and engaged less in social sniffing and allogrooming behaviors. While social approach behavior in response to 50-kHz USV playback was evident in male but not female *Shank3* deficient rats, male *Shank3* deficient rats did not display search behavior after the playback presentation ended [100]. More prominent deficits were evident in *Ube3a3* deficient rats [102]. In *Ube3a3* deficient rats, the response to 50-kHz USV playback was clearly reduced, as reflected in a less prominent increase in locomotor activity, lower levels of social approach behavior during 50-kHz USV playback, and lack of search behavior after the playback presentation ended. In humans, *UBE3A* deletions cause Angelman syndrome characterized by severe developmental delay and intellectual disability, most notably lack of language acquisition [101]. Finally, we found that environmental risk factors associated with neurodevelopmental disorders, most notably early life exposure to the organophosphorus pesticide chlorpyrifos, reduced social approach behavior in response to 50-kHz USV playback [103]. Specifically, chlorpyrifos exposure led to a dose-dependent inhibition of social approach behavior, with the two highest doses completely blocking the social approach response in females but not males. Together, this indicates that measuring social approach behavior evoked by playback of 50-kHz USV in the receiver can help to reveal social behavior and ultrasonic communication deficits in genetic and environmental models with relevance to human neuropsychiatric dysfunctions.

## 4. Social Behavior and Ultrasonic Vocalizations in *Cacna1c* Haploinsufficient Rats

As part of a translational effort to better understand major genetic risk factors for neuropsychiatric dysfunctions [104], we applied a longitudinal deep phenotyping approach and contributed to the behavioral characterization of a genetic rat model haploinsufficient for the cross-disorder risk gene *Cacna1c*. Because deficits in processing social signals are associated with reduced social functioning as commonly seen in neuropsychiatric disorders, we focused on socio-affective communication through 22-kHz and 50-kHz USV. In our studies, we compared constitutive heterozygous *Cacna1c^+/−^* rats to wildtype *Cacna1c^+/+^* littermate controls [15,16,17,18,19].

In our first study, we focused on juvenile male rats [15]. We applied a reciprocal approach for studying socio-affective communication in sender and receiver by including rough-and-tumble play and playback of 50-kHz USV. 

Firstly, we compared rough-and-tumble play and concomitant 50-kHz USV emission between *Cacna1c^+/−^* rats and *Cacna1c^+/+^* littermate controls. Rough-and-tumble play was not affected by *Cacna1c* haploinsufficiency in juvenile male rats. Specifically, the total time spent playing did not differ between genotypes. Moreover, the occurrence of specific components of the rough-and-tumble play repertoire, such as pinning, wrestling, and chasing, was not affected. 

**Figure 2:**
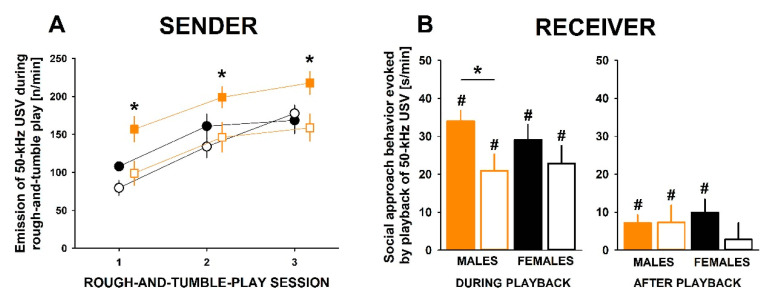
Effects of *Cacna1c* haploinsufficiency on (**A**) the emission of 50-kHz ultrasonic vocalizations (USV) emitted during rough-and-tumble play in the sender (filled orange squares = male *Cacna1c^+/+^* littermate controls; open orange squares = male *Cacna1c^+/−^* rats; filled black circles = female *Cacna1c^+/+^* littermate controls; open white circles = female *Cacna1c^+/−^* rats; *N* = 10 play pairs each) and (**B**) on social approach behavior evoked by playback of 50-kHz USV in the receiver (filled orange bars = male *Cacna1c^+/+^* littermate controls; open orange bars = male *Cacna1c^+/−^* rats; filled black bars = female *Cacna1c^+/+^* littermate controls; open black bars = female *Cacna1c^+/−^* rats; *N* = 20 rats each). Please note that social approach behavior is shown as change scores from baseline. Data were previously reported in [15,16]. * *p* < 0.05 vs. male *Cacna1c^+/+^* littermate controls; ^#^ *p* < 0.05 vs. baseline.

The emission of 50-kHz USV, however, was strongly affected by *Cacna1c* haploinsufficiency in juvenile male rats (Figure 2A). *Cacna1c^+/−^* rats emitted fewer 50-kHz USV than *Cacna1c^+/+^* littermate controls. This genotype difference was evident during actual play phases but also during non-playful social interactions. While 50-kHz USV emission was particularly high during wrestling and chasing in *Cacna1c^+/+^* littermate controls, this was not the case in *Cacna1c^+/−^* rats where high 50-kHz USV emission rates were exclusively seen during chasing. 

Moreover, *Cacna1c* haploinsufficiency affected certain 50-kHz USV subtypes more than others and the overall reduction in 50-kHz USV was primarily driven by reduced numbers of FLAT and MIXED calls. Related to that, there were prominent genotype effects on the prevalence of 50-kHz USV subtypes during specific components of the rough-and-tumble play repertoire. During wrestling and chasing, TRILL calls were increased in *Cacna1c^+/−^* rats as compared to *Cacna1c^+/+^* littermate controls, primarily at the cost of MIXED calls. No prominent differences were seen during pinning. Finally, acoustic features of 50-kHz USV differed between genotypes. Most notably, the peak frequency of 50-kHz USV was higher in *Cacna1c^+/−^* rats than *Cacna1c^+/+^* littermate controls. Peak amplitude was lower in *Cacna1c^+/−^* rats, while call duration and frequency modulation were not affected by *Cacna1c* haploinsufficiency.

Secondly, we measured social approach behavior evoked by the playback of 50-kHz USV (Figure 2B). Although both genotypes displayed a social preference, social approach behavior was reduced in *Cacna1c^+/−^* rats, as compared to *Cacna1c^+/+^* littermate controls. Moreover, *Cacna1c^+/+^* littermate controls but not *Cacna1c^+/−^* rats displayed search behavior after the playback presentation ended. No increase in locomotor activity was evident during and after playback. 

Together, the first study indicates that a prominent reduction of Cav1.2 expression in the brain has detrimental effects on socio-affective communication through 50-kHz USV in male rats haploinsufficient for the cross-disorder risk gene *Cacna1c* [15]. Importantly, *Cacna1c* haploinsufficiency affects not only the emission of 50-kHz USV in the sender but also socio-affective information processing in the receiver and leads to an altered response to 50-kHz USV serving an affiliative function as social contact calls. Given that 50-kHz USV emission was associated with positive affect, this indicates that *Cacna1c^+/−^* rats experience rough-and-tumble play as less rewarding, and it would be interesting to test whether this is reflected in lower levels of social conditioned place preference [105]. 

In our second study, we focused on juvenile female rats [16,17]. The females in this second study were littermates of the male rats used in the first study and were tested in the exact same behavioral paradigms [15]. This gave us the opportunity to compare males and females and to test whether the effects of *Cacna1c* haploinsufficiency are modulated by sex. 

In contrast to males, *Cacna1c* haploinsufficiency exerted strong effects on rough-and-tumble play in females [16]. Specifically, the total time spent playing was clearly higher in female *Cacna1c^+/−^* rats as compared to female *Cacna1c^+/+^* littermate controls. In fact, female *Cacna1c^+/−^* rats engaged even more in rough-and-tumble play than male *Cacna1c^+/+^* littermate controls. This is particularly remarkable given the typical sex difference in rough-and-tumble play, with male rats playing more than females [31]. The increase in rough-and-tumble play was driven by remarkably high levels of pinning behavior [16]. Pinning behavior in *Cacna1c^+/−^* rats was not only higher than in female *Cacna1c^+/+^* littermate controls but also higher than in male *Cacna1c^+/+^* littermate controls. No prominent genotype effects on wrestling and chasing were found. 

The increase in rough-and-tumble play behavior displayed by female *Cacna1c^+/−^* rats, however, was not paralleled by an increase in the 50-kHz USV emission rate (Figure 2A). In fact, female *Cacna1c^+/−^* rats emitted a similarly high number of 50-kHz USV as female *Cacna1c^+/+^* littermate controls and slightly less than male *Cacna1c^+/+^* littermate controls [16]. Although the 50-kHz USV emission rate was not affected by *Cacna1c* haploinsufficiency, the temporal organization of 50-kHz USV emission was [17]. Similar to male *Cacna1c^+/+^* littermate controls, female *Cacna1c^+/−^* rats emitted the highest numbers of 50-kHz USV during actual play phases as opposed to non-playful social interactions. Such a close association with the actual play phase was not seen in female *Cacna1c^+/+^* littermate controls. Moreover, again similar to male *Cacna1c^+/+^* littermate controls, the emission of 50-kHz USV was highest during wrestling and chasing in female *Cacna1c^+/−^* rats. Once more, such a close association with specific components of the rough-and-tumble play repertoire, however, was not seen in female *Cacna1c^+/+^* littermate controls. 

Moreover, *Cacna1c* haploinsufficiency had minor effects on 50-kHz USV subtypes [17]. Female *Cacna1c^+/−^* rats emitted more STEP calls than female *Cacna1c^+/+^* littermate controls. Likewise, the number of overlapping calls seen when two rats emitted 50-kHz USV at the same time was higher in female *Cacna1c^+/−^* rats. In fact, female *Cacna1c^+/−^* rats but not female *Cacna1c^+/+^* littermate controls reached levels seen in male *Cacna1c^+/+^* littermate controls. However, there were no prominent genotype effects on the prevalence of 50-kHz USV subtypes during specific components of the rough-and-tumble play repertoire. Acoustic features of 50-kHz USV did not differ between genotypes. 

When measuring social approach behavior evoked by playback of 50-kHz USV in females, both genotypes displayed an increase in locomotor activity with a clear social preference (Figure 2A). No genotype differences were evident during playback [16]. After the playback presentation ended, however, female *Cacna1c^+/+^* littermate controls but not female *Cacna1c^+/−^* rats displayed search behavior, similar to the pattern in males. No increase in locomotor activity was evident after playback. Response calls evoked by the playback of 50-kHz USV occurred in *Cacna1c^+/−^* rats and *Cacna1c^+/+^* littermate controls of both sexes, although their emission was found to be particularly low in female *Cacna1c^+/−^* rats [16].

Together, the second study indicates that the effects of *Cacna1c* haploinsufficiency are modulated by sex. Most notably, *Cacna1c* haploinsufficiency in females led to hypermasculinization of rough-and-tumble play, as reflected in a clear increase in pinning behavior, without exerting prominent effects on the emission of 50-kHz USV [16,17]. Because sex differences in rough-and-tumble play were repeatedly associated with differences in testosterone levels [106], it would be interesting to compare *Cacna1c^+/−^* rats and *Cacna1c^+/+^* littermate controls of both sexes and to quantify testosterone. 

In the third study, we focused on social interactions in adult female rats [18]. In contrast to the studies in juvenile rats, this included not only same-genotype dyads but also mixed-genotype dyads. Through this means, we showed that the emission of 50-kHz USV is highest in dyads consisting of two *Cacna1c^+/+^* littermate controls, but lowest in dyads with two *Cacna1c^+/−^* rats. Intermediate levels of 50-kHz USV were seen in mixed-genotype dyads with one *Cacna1c^+/−^* rat and a *Cacna1c^+/+^* littermate control. 

During social interactions, all major 50-kHz USV subtypes occurred. FLAT and TRILL calls were most prevalent, while STEP calls occurred rarely. *Cacna1c* haploinsufficiency had no prominent effect on their prevalence. Acoustic features did not differ between genotypes, with the exception of peak amplitude, which was lower in dyads including one or two *Cacna1c^+/−^* rats, particularly for FLAT calls.

In line with the hypermasculinized rough-and-tumble play behavior displayed by female *Cacna1c^+/−^* rats, *Cacna1c^+/−^* rats behaved in a more dominant manner in the tube test. During social interactions, however, aggressive behavior was rarely seen and not enhanced in dyads with one or two *Cacna1c^+/−^* rats. In fact, there were no effects of *Cacna1c* haploinsufficiency on social behavior at the level of dyads, apart from increased physical contact in *Cacna1c^+/−^* rats. Interestingly, however, non-social behaviors were affected. Rearing and digging were reduced in dyads including *Cacna1c^+/−^* rats, while self-grooming behavior was strongly enhanced.

Detailed temporal analyses using synchronized high-resolution recordings revealed that the emission of 50-kHz USV was higher during social behaviors than non-social behaviors. Particularly high 50-kHz USV emission rates occurred during following behavior. Moderate levels of 50-kHz USV were emitted during social sniffing and physical contact. Lowest numbers of 50-kHz USV were seen during social grooming. In fact, 50-kHz USV emission during social grooming was not higher than during self-grooming in *Cacna1c^+/+^* littermate controls.

The reduced emission of 50-kHz USV in dyads including *Cacna1c^+/−^* rats was seen during social behaviors and non-social behaviors. The genotype effects were most prominent during following behavior, but also seen during rearing, digging, and self-grooming, suggesting that the enhanced level of self-grooming in dyads including *Cacna1c^+/−^* rats was not associated with positive affect reflected by 50-kHz USV.

We further took advantage of the mixed-genotype dyads and individually analyzed the behavior of *Cacna1c^+/−^* rats and *Cacna1c^+/+^* littermate controls while socially interacting with the other genotype. This detailed analysis revealed that *Cacna1c^+/+^* littermate controls spent more time sniffing the partner than *Cacna1c^+/−^* rats, indicating that *Cacna1c^+/−^* rats were extensively sniffed by *Cacna1c^+/+^* littermate controls but did not reciprocate. When comparing the behavioral profile displayed by *Cacna1c^+/−^* rats during a social interaction with other *Cacna1c^+/−^* rats and *Cacna1c^+/+^* littermate controls, the level of physical contact in *Cacna1c^+/−^* rats was higher while socially interacting with other *Cacna1c^+/−^* rats. In *Cacna1c^+/+^* littermate controls, their social behavior was not affected by the genotype of the partner. 

Together, the third study indicates that effects of *Cacna1c* haploinsufficiency on social behavior and socio-affective communication through 50-kHz USV are also evident in adult rats. Most notably, detailed temporal analyses revealed prominent reductions in 50-kHz USV during social but also non-social behaviors, suggesting that their reduced emission in *Cacna1c^+/−^* rats is not specifically linked to deficits in social behavior. Considering the effects of *Cacna1c* haploinsufficiency on dominance behavior, it would be interesting to test how the presence of *Cacna1c^+/−^* rats affects aggressive behavior and the social hierarchy in mixed-genotype groups of rats [107]. 

Finally, in our fourth study, we applied a recently refined 22-kHz USV playback paradigm [46] and tested whether *Cacna1c* haploinsufficiency leads to an altered response to 22-kHz USV serving an alarming function [19]. As expected, male and female *Cacna1c^+/+^* littermate controls displayed behavioral inhibition in response to playback of 22-kHz USV. Behavioral inhibition evoked by 22-kHz USV was evident in response to 22-kHz USV recorded during both predator urine exposure and a retention test on learned fear. The lack of prominent differences in the potency to elicit behavioral inhibition depending on the threat context used for recording 22-kHz USV supports the generalizability of their alarming effects. However, generalizability was limited in *Cacna1c^+/−^* rats in a sex-dependent manner and *Cacna1c* haploinsufficiency led to less pronounced and less specific behavioral inhibition in male but not female rats. 

Together, the fourth study shows that behavioral inhibition evoked by playback of alarm 22-kHz USV is robust and occurs in response to both sets of 22-kHz USV yet is modulated by *Cacna1c* in a sex-dependent manner. Considering the less pronounced and less specific behavioral inhibition evoked by 22-kHz USV in *Cacna1c^+/−^* rats, it would be interesting to test whether *Cacna1c* haploinsufficiency affects the emission of 22-kHz USV in response to predator exposure [45] or during fear learning [108]. 

In summary, the four studies indicate that *Cacna1c* haploinsufficiency in rats leads to robust deficits in socio-affective communication through 22-kHz and 50-kHz USV. *Cacna1c* haploinsufficiency affected the sender in a sex-specific way. In males, *Cacna1c* haploinsufficiency led to reduced 50-kHz USV emission during rough-and-tumble play. In females, *Cacna1c* haploinsufficiency led to hypermasculinization of the rough-and-tumble-play repertoire as juveniles and lower emission of 50-kHz USV and mild alterations in social behavior in adulthood. *Cacna1c* haploinsufficiency also affected the receiver. Social approach behavior evoked by 50-kHz USV was reduced in both male and female *Cacna1c* haploinsufficient rats, although effects were more prominent in males. Moreover, male but not female *Cacna1c* haploinsufficient displayed less pronounced and less specific behavioral inhibition evoked by 22-kHz USV. *Cacna1c* haploinsufficiency is thus associated with a variety of alterations in social behavior, possibly due to lower motivation and/or diminished capability to display appropriate responses to important socio-affective communication signals.

## 5. Other Behavioral Phenotypes Displayed by *Cacna1c* Haploinsufficient Rats

As compared to the prominent effects of *Cacna1c* haploinsufficiency on socio-affective communication through 22-kHz and 50-kHz USV, reduced expression of Cav1.2 had only moderate effects in our studies on learning and memory. Our studies revealed intact spatial memory and reversal learning capabilities in a radial arm maze using food as reward, with slightly superior memory performance at the cost of reduced cognitive flexibility in *Cacna1c^+/−^* rats [13]. Such effects were primarily seen in males but not females. Social and physical enrichment had positive effects in *Cacna1c^+/−^* rats and *Cacna1c^+/+^* littermate controls and ameliorated slight reversal learning deficits displayed by *Cacna1c^+/−^* rats [14]. Novel object recognition memory was not affected by *Cacna1c* haploinsufficiency but impaired following post-weaning social isolation in both genotypes. 

In an independent series of experiments performed by another laboratory, touchscreens were used for reversal learning with sucrose solution as reward. While learning was intact, *Cacna1c^+/−^* rats were found to be impaired in reversal learning and made more errors during reversal than *Cacna1c^+/+^* littermate controls [109]. In a related study, the effects of *Cacna1c* haploinsufficiency on delay and trace auditory fear conditioning was tested [110]. In the delay condition that typically results in strong conditioning to the auditory cue, *Cacna1c^+/−^* rats were found to display an increased fear response to the context. In the trace condition that typically leads to strong conditioning to the context, however, *Cacna1c^+/−^* rats displayed the opposite pattern and showed an elevated fear response to the auditory cue. In an unpaired condition, the fear response to both auditory cue and context was enhanced in *Cacna1c^+/−^* rats and it was suggested that *Cacna1c* haploinsufficiency is associated with inappropriate fear responses. 

In the most recent study performed by the other laboratory, a contextual fear conditioning paradigm was applied, and contextual fear memory was found to be unchanged in *Cacna1c^+/−^* rats [107]. However, following a pre-exposure to the to-be-conditioned context, *Cacna1c^+/−^* rats displayed a much stronger fear response than *Cacna1c^+/+^* littermate controls, which was interpreted as evidence for impaired latent inhibition in *Cacna1c^+/−^* rats. 

## 6. Neurobiological Alterations in *Cacna1c* Haploinsufficient Rats

At the neurobiological level, we obtained evidence for *Cacna1c* playing an important role in mitochondrial integrity and function [22] by showing that *Cacna1c* downregulation promotes resilience against glutamate-induced oxidative stress in neurons [21]. However, *Cacna1c* haploinsufficiency did not affect mitochondrial bioenergetics and reactive oxygen species production in rats and this was independent of whether rats were exposed to post-weaning social isolation or social and physical enrichment [20]. We also did not obtain evidence for the effects of *Cacna1c* haploinsufficiency on brain morphology by comparing volumetric properties of hippocampus and prefrontal cortex [23]. Likewise, adult hippocampal neurogenesis was not affected by *Cacna1c* haploinsufficiency. When quantifying cell proliferation and survival through an immunofluorescent multiple staining approach to ensure neuronal cell type specificity, no genotype differences were seen [23]. In another study, however, reduced cell proliferation in *Cacna1c^+/−^* rats was reported, albeit in absence of effects of *Cacna1c* haploinsufficiency on immature neurons and the size of the dentate gyrus [111]. *Cacna1c* haploinsufficiency was also reported to be associated with reduced brain-derived neurotrophic factor (BDNF) expression in the prefrontal cortex [109].

In the most recent study on impaired latent inhibition during contextual fear conditioning in *Cacna1c^+/−^* rats, neurobiological mechanisms implicated in learning and memory were studied by focusing on the dorsal hippocampus [112]. Specifically, associative plasticity at CA1 pyramidal synapses and network synchronization through phase-amplitude coupling between theta and gamma oscillations of the CA1 local field potential were studied. It was shown that synaptic plasticity is affected by *Cacna1c* haploinsufficiency and that the induction of long-term potentiation through theta-burst pairing is impaired in *Cacna1c^+/−^* rats. It was further found that spine calcium signaling is impaired during postsynaptic spike bursts in CA1 pyramidal neurons form *Cacna1c^+/−^* rats. Moreover, phase-amplitude coupling during exploration of a novel environment was reduced in *Cacna1c^+/−^* rats in the absence of behavioral differences in the novelty response. Genotypes did not differ in a familiar environment. 

Similar to previous studies [23,111], hippocampal morphology and cellular density did not differ between *Cacna1c^+/−^* rats and *Cacna1c^+/+^* littermate controls [112]. However, phosphorylated extracellular-signal regulated kinase (pERK) and cAMP response element-binding protein (pCREB) immunoreactivities were significantly reduced in the hippocampus of *Cacna1c^+/−^* rats, without genotype effects on total ERK and CREB levels. It was thus suggested that impaired ERK- and CREB-mediated synapse-to-nucleus signaling in *Cacna1c^+/−^* rats might contribute to hippocampal dysfunctions, eventually translating into impairments in learning and memory. This view is supported by the observation that activation of ERK signaling through the BDNF mimetic TrkB/TrkC neurotrophin receptor co-activator LM22B-10 restored pERK and pCREB levels and led to a reversal of the long-term potentiation deficit in *Cacna1c^+/−^* rats. Most importantly, intra-hippocampal administration of LM22B-10 treatment also reversed the impairment in latent inhibition during contextual fear conditioning in *Cacna1c^+/−^* rats. A similar effect was seen following an intra-peritoneal injection of LM22B-10. Together, this suggests that impaired ERK signaling-mediated excitation-transcription coupling underlies the learning and memory deficits seen in *Cacna1c^+/−^* rats.

## 7. Conclusions

We conclude that *Cacna1c* haploinsufficiency in rats leads to robust deficits in socio-affective communication through 22-kHz and 50-kHz USV and associated alterations in social behavior, such as rough-and-tumble play behavior (Table 1). These deficits appear to be more severe than related deficits displayed by established rat models for neurodevelopmental disorders, such as the *Shank3* deficient rat model for autism. However, no neurobiological correlate has been identified yet. Comparatively mild effects of *Cacna1c* haploinsufficiency on learning and memory were reported. Such deficits were linked to impaired ERK signaling.

## 8. Future Perspectives

Because socio-affective communication through 22-kHz and 50-kHz USV is involved in the regulation of many social behaviors, it would be interesting to see whether the effects of *Cacna1c* haploinsufficiency on sender and receiver affect other social behaviors, such as cooperative actions [36], mutual rewarding preferences [37], general and direct reciprocity [38], and empathy-driven helping behavior [39]. For instance, it was suggested that 50-kHz USV emission is involved in cooperative behavior in an instrumental task [113]. Related to that, it would be interesting to test whether the reduction in 50-kHz USV emission in *Cacna1c^+/−^* rats occurs specifically in a social context or whether this is also seen in response to non-social stimuli. Because *CACNA1C* SNPs are strongly associated with affective disorders, namely depression [1] and bipolar disorder [2], in humans, it would be interesting to measure mania-like elevated mood through amphetamine-induced 50-kHz USV in *Cacna1c^+/−^* rats. Amphetamine is known to be a very potent elicitor of 50-kHz USV in rats [114]. Finally, given the evidence in support of the notion that impaired ERK signaling-mediated excitation-transcription coupling underlies the learning and memory deficits seen in *Cacna1c^+/−^* rats, it would be interesting to test whether impaired ERK signaling is associated with the deficits in socio-affective communication through 22-kHz and 50-kHz USV and whether associated alterations in social behavior can be similarly rescued by a BDNF mimetic, such as LM22B-10.

## Figures and Tables

**Table 1:** Effects of *Cacna1c* haploinsufficiency on ultrasonic communication in rats.

Sender	Receiver	Sex	Age	References
Reduced 50-kHz USV emission rates during rough-and-tumble play	Reduced social approach behavior evoked by playback of pro-social 50-kHz USV	Male	Juvenility	[15]
Unchanged 50-kHz USV emission rates during rough-and-tumble play	Unchanged social approach behavior evoked by playback of pro-social 50-kHz USV ^1^	Female	Juvenility	[16,17]
Reduced 50-kHz USV emission rates during social interactions		Female	Adulthood	[18]
	Reduced behavioral inhibition evoked by playback of alarm 22-kHz USV	Male	Adulthood	[19]
	Unchanged behavioral inhibition evoked by playback of alarm 22-kHz USV	Female	Adulthood	[19]

USV = ultrasonic vocalizations; ^1^ Social approach behavior evoked during 50-kHz USV playback was unchanged, yet search behavior after playback was reduced.

## References

[1] Green E.K., Grozeva D., Jones I., Jones L., Kirov G., Caesar S., Gordon-Smith K., Fraser C., Forty L., Russell E. (2010). The bipolar disorder risk allele at CACNA1C also confers risk of recurrent major depression and of schizophrenia. Mol. Psychiatry.

[2] Ferreira M.A., O’Donovan M.C., Meng Y.A., Jones I.R., Ruderfer D.M., Jones L., Fan J., Kirov G., Perlis R.H., Green E.K. (2008). Collaborative genome-wide association analysis supports a role for ANK3 and CACNA1C in bipolar disorder. Nat. Genet..

[3] Splawski I., Timothy K.W., Sharpe L.M., Decher N., Kumar P., Bloise R., Napolitano C., Schwartz P.J., Joseph R.M., Condouris K. (2004). Ca(V)1.2 calcium channel dysfunction causes a multisystem disorder including arrhythmia and autism. Cell.

[4] Nyegaard M., Demontis D., Foldager L., Hedemand A., Flint T.J., Sørensen K.M., Andersen P.S., Nordentoft M., Werge T., Pedersen C.B. (2010). CACNA1C (rs1006737) is associated with schizophrenia. Mol. Psychiatry.

[5] Zamponi G.W. (2016). Targeting voltage-gated calcium channels in neurological and psychiatric diseases. Nat. Rev. Drug Discov..

[6] Kabir Z.D., Lee A.S., Rajadhyaksha A.M. (2016). L-type Ca^2+^ channels in mood, cognition and addiction: Integrating human and rodent studies with a focus on behavioural endophenotypes. J. Physiol..

[7] Bigos K.L., Mattay V.S., Callicott J.H., Straub R.E., Vakkalanka R., Kolachana B., Hyde T.M., Lipska B.K., Kleinman J.E., Weinberger D.R. (2010). Genetic variation in CACNA1C affects brain circuitries related to mental illness. Arch. Gen. Psychiatry.

[8] Eckart N., Song Q., Yang R., Wang R., Zhu H., McCallion A.S., Avramopoulos D. (2016). Functional Characterization of Schizophrenia-Associated Variation in CACNA1C. PLoS ONE.

[9] Gershon E.S., Grennan K., Busnello J., Badner J.A., Ovsiew F., Memon S., Alliey-Rodriguez N., Cooper J., Romanos B., Liu C. (2014). A rare mutation of CACNA1C in a patient with bipolar disorder, and decreased gene expression associated with a bipolar-associated common SNP of CACNA1C in brain. Mol. Psychiatry.

[10] Jaffe A.E., Hoeppner D.J., Saito T., Blanpain L., Ukaigwe J., Burke E.E., Collado-Torres L., Tao R., Tajinda K., Maynard K.R. (2020). Profiling gene expression in the human dentate gyrus granule cell layer reveals insights into schizophrenia and its genetic risk. Nat. Neurosci..

[11] Roussos P., Mitchell A.C., Voloudakis G., Fullard J.F., Pothula V.M., Tsang J., Stahl E.A., Georgakopoulos A., Ruderfer D.M., Charney A. (2014). A role for noncoding variation in schizophrenia. Cell Rep..

[12] Yoshimizu T., Pan J.Q., Mungenast A.E., Madison J.M., Su S., Ketterman J., Ongur D., McPhie D., Cohen B., Perlis R. (2015). Functional implications of a psychiatric risk variant within CACNA1C in induced human neurons. Mol. Psychiatry.

[13] Braun M.D., Kisko T.M., Vecchia D.D., Andreatini R., Schwarting R.K.W., Wöhr M. (2018). Sex-specific effects of Cacna1c haploinsufficiency on object recognition, spatial memory, and reversal learning capabilities in rats. Neurobiol. Learn. Mem..

[14] Braun M.D., Kisko T.M., Witt S.H., Rietschel M., Schwarting R.K.W., Wöhr M. (2019). Long-term environmental impact on object recognition, spatial memory and reversal learning capabilities in Cacna1c-haploinsufficient rats. Hum. Mol. Genet..

[15] Kisko T.M., Braun M.D., Michels S., Witt S.H., Rietschel M., Culmsee C., Schwarting R.K.W., Wöhr M. (2018). Cacna1c haploinsufficiency leads to pro-social 50-kHz ultrasonic communication deficits in rats. Dis. Models Mech..

[16] Kisko T.M., Braun M.D., Michels S., Witt S.H., Rietschel M., Culmsee C., Schwarting R.K.W., Wöhr M. (2020). Sex-dependent effects of Cacna1c haploinsufficiency on juvenile social play behavior and pro-social 50-kHz ultrasonic communication in rats. Genes Brain Behav..

[17] Kisko T.M., Schwarting R.K.W., Wöhr M. (2021). Sex differences in the acoustic features of social play-induced 50-kHz ultrasonic vocalizations: A detailed spectrographic analysis in wild-type Sprague-Dawley and Cacna1c haploinsufficient rats. Dev. Psychobiol..

[18] Redecker T.M., Kisko T.M., Schwarting R.K.W., Wöhr M. (2019). Effects of Cacna1c haploinsufficiency on social interaction behavior and 50-kHz ultrasonic vocalizations in adult female rats. Behav. Brain Res..

[19] Wöhr M., Willadsen M., Kisko T.M., Schwarting R.K.W., Fendt M. (2020). Sex-dependent effects of Cacna1c haploinsufficiency on behavioral inhibition evoked by conspecific alarm signals in rats. Prog. Neuro-Psychopharmacol. Biol. Psychiatry.

[20] Michels S., Dolga A.M., Braun M.D., Kisko T.M., Sungur A.Ö., Witt S.H., Rietschel M., Dempfle A., Wöhr M., Schwarting R.K.W. (2019). Interaction of the psychiatric risk gene Cacna1c with post-weaning social isolation or environmental enrichment does not affect brain mitochondrial bioenergetics in rats. Front. Cell Neurosci..

[21] Michels S., Ganjam G.K., Martins H., Schratt G.M., Wöhr M., Schwarting R.K.W., Culmsee C. (2018). Downregulation of the psychiatric susceptibility gene Cacna1c promotes mitochondrial resilience to oxidative stress in neuronal cells. Cell Death Discov..

[22] Michels S., Wöhr M., Schwarting R.K.W., Culmsee C. (2018). Psychiatric risk gene Cacna1c determines mitochondrial resilience against oxidative stress in neurons. Cell Death Discov..

[23] Redecker T.M., Kisko T.M., Wöhr M., Schwarting R.K.W. (2020). Cacna1c haploinsufficiency lacks effects on adult hippocampal neurogenesis and volumetric properties of prefrontal cortex and hippocampus in female rats. Physiol. Behav..

[24] Krautheim J.T., Straube B., Dannlowski U., Pyka M., Schneider-Hassloff H., Drexler R., Krug A., Sommer J., Rietschel M., Witt S.H. (2018). Outgroup emotion processing in the vACC is modulated by childhood trauma and CACNA1C risk variant. Soc. Cogn. Affect. Neurosci..

[25] Nieratschker V., Brückmann C., Plewnia C. (2015). CACNA1C risk variant affects facial emotion recognition in healthy individuals. Sci. Rep..

[26] Soeiro-de-Souza M.G., Otaduy M.C., Dias C.Z., Bio D.S., Machado-Vieira R., Moreno R.A. (2012). The impact of the CACNA1C risk allele on limbic structures and facial emotions recognition in bipolar disorder subjects and healthy controls. J. Affect. Disord..

[27] Krug A., Nieratschker V., Markov V., Krach S., Jansen A., Zerres K., Eggermann T., Stöcker T., Shah N.J., Treutlein J. (2010). Effect of CACNA1C rs1006737 on neural correlates of verbal fluency in healthy individuals. Neuroimage.

[28] Schweinfurth M.K. (2020). The social life of Norway rats (*Rattus norvegicus*). eLife.

[29] Lukas M., de Jong T.R. (2017). Conspecific interactions in adult laboratory rodents: Friends or foes?. Curr. Top. Behav. Neurosci..

[30] Vanderschuren L.J., Achterberg E.J., Trezza V. (2016). The neurobiology of social play and its rewarding value in rats. Neurosci. Biobehav. Rev..

[31] Pellis S.M., Field E.F., Smith L.K., Pellis V.C. (1997). Multiple differences in the play fighting of male and female rats. Implications for the causes and functions of play. Neurosci. Biobehav. Rev..

[32] Trezza V., Baarendse P.J., Vanderschuren L.J. (2010). The pleasures of play: Pharmacological insights into social reward mechanisms. Trends Pharmacol. Sci..

[33] Perna J.C., Engelmann M. (2017). Recognizing others: Rodent’s social memories. Curr. Top. Behav. Neurosci..

[34] Galef B.G., Wigmore S.W. (1983). Transfer of information concerning distant foods: A laboratory investigation of the ‘information-centre’ hypothesis. Anim. Behav..

[35] Fendt M., Gonzalez-Guerrero C.P., Kahl E. (2021). Observational fear learning in rats: Role of trait anxiety and ultrasonic vocalization. Brain Sci..

[36] Avital A., Aga-Mizrachi S., Zubedat S. (2016). Evidence for social cooperation in rodents by automated maze. Sci. Rep..

[37] Hernandez-Lallement J., van Wingerden M., Marx C., Srejic M., Kalenscher T. (2015). Rats prefer mutual rewards in a prosocial choice task. Front. Neurosci..

[38] Rutte C., Taborsky M. (2007). Generalized reciprocity in rats. PLoS Biol..

[39] Bartal I.B.A., Decety J., Mason P. (2011). Empathy and pro-social behavior in rats. Science.

[40] Brudzynski S.M. (2013). Ethotransmission: Communication of emotional states through ultrasonic vocalization in rats. Curr. Opin. Neurobiol..

[41] Wöhr M., Schwarting R.K.W. (2013). Affective communication in rodents: Ultrasonic vocalizations as a tool for research on emotion and motivation. Cell Tissue Res..

[42] Brudzynski S.M. (2021). Biological functions of rat ultrasonic vocalizations, Arousal mechanisms and call initiation. Brain Sci..

[43] Hofer M.A. (1996). Multiple regulators of ultrasonic vocalization in the infant rat. Psychoneuroendocrinology.

[44] Allin J.T., Banks E.M. (1972). Functional aspects of ultrasound production by infant albino rats (Rattus norvegicus). Anim. Behav..

[45] Blanchard R.J., Blanchard D.C., Agullana R., Weiss S.M. (1991). Twenty-two kHz alarm cries to presentation of a predator, by laboratory rats living in visible burrow systems. Physiol. Behav..

[46] Fendt M., Brosch M., Wernecke K.E.A., Willadsen M., Wöhr M. (2018). Predator odour but not TMT induces 22-kHz ultrasonic vocalizations in rats that lead to defensive behaviours in conspecifics upon replay. Sci. Rep..

[47] Knutson B., Burgdorf J., Panksepp J. (1998). Anticipation of play elicits high-frequency ultrasonic vocalizations in young rats. J. Comp. Psychol..

[48] Sales G.D. (1972). Ultrasound and mating behaviour in rodents with some observations on other behavioural situations. J. Zool..

[49] Panksepp J. (2005). Psychology. Beyond a joke: From animal laughter to human joy?. Science.

[50] Burgdorf J., Panksepp J., Moskal J.R. (2011). Frequency-modulated 50 kHz ultrasonic vocalizations: A tool for uncovering the molecular substrates of positive affect. Neurosci. Biobehav. Rev..

[51] Pereira M., Andreatini R., Schwarting R.K.W., Brenes J.C. (2014). Amphetamine-induced appetitive 50-kHz calls in rats: A marker of affect in mania?. Psychopharmacology.

[52] Wright J.M., Gourdon J.C., Clarke P.B. (2010). Identification of multiple call categories within the rich repertoire of adult rat 50-kHz ultrasonic vocalizations: Effects of amphetamine and social context. Psychopharmacology.

[53] Wöhr M. (2018). Ultrasonic communication in rats: Appetitive 50-kHz ultrasonic vocalizations as social contact calls. Behav. Ecol. Sociobiol..

[54] Berridge K.C., Robinson T.E., Aldridge J.W. (2009). Dissecting components of reward: ‘liking’, ‘wanting’, and learning. Curr. Opin. Pharmacol..

[55] Burke C.J., Modlinska K., Mauro M.H., Aleksandrova L.R., Pellis S.M., Phillips A.G., Euston D.R. (2021). A naturalistic method to test depression: Anticipation of play. Behav. Brain Res..

[56] Burgdorf J., Kroes R.A., Moskal J.R., Pfaus J.G., Brudzynski S.M., Panksepp J. (2008). Ultrasonic vocalizations of rats (Rattus norvegicus) during mating, play, and aggression: Behavioral concomitants, relationship to reward, and self-administration of playback. J. Comp. Psychol..

[57] Himmler B.T., Kisko T.M., Euston D.R., Kolb B., Pellis S.M. (2014). Are 50-kHz calls used as play signals in the playful interactions of rats? I. Evidence from the timing and context of their use. Behav. Process..

[58] Manduca A., Campolongo P., Palmery M., Vanderschuren L.J., Cuomo V., Trezza V. (2014). Social play behavior, ultrasonic vocalizations and their modulation by morphine and amphetamine in Wistar and Sprague-Dawley rats. Psychopharmacology.

[59] Manduca A., Servadio M., Campolongo P., Palmery M., Trabace L., Vanderschuren L.J., Cuomo V., Trezza V. (2014). Strain-and context-dependent effects of the anandamide hydrolysis inhibitor URB597 on social behavior in rats. Eur. Neuropsychopharmacol..

[60] Siviy S.M., Panksepp J. (1987). Sensory modulation of juvenile play in rats. Dev. Psychobiol..

[61] Kisko T.M., Euston D.R., Pellis S.M. (2015). Are 50-kHz calls used as play signals in the playful interactions of rats? III. The effects of devocalization on play with unfamiliar partners as juveniles and as adults. Behav. Process..

[62] Kisko T.M., Himmler B.T., Himmler S.M., Euston D.R., Pellis S.M. (2015). Are 50-kHz calls used as play signals in the playful interactions of rats? II. Evidence from the effects of devocalization. Behav. Process..

[63] Kisko T.M., Wöhr M., Pellis V.C., Pellis S.M. (2017). From Play to Aggression: High-Frequency 50-kHz Ultrasonic Vocalizations as Play and Appeasement Signals in Rats. Curr. Top. Behav. Neurosci..

[64] Panksepp J., Burgdorf J. (2000). 50-kHz chirping (laughter?) in response to conditioned and unconditioned tickle-induced reward in rats: Effects of social housing and genetic variables. Behav. Brain Res..

[65] Panksepp J., Burgdorf J., Gordon N., Kazniak A. (2001). Towards a genetics of joy: Breeding rats for “laughter”. Emotions, Qualia, and Consciousness.

[66] Burgdorf J., Panksepp J., Brudzynski S.M., Beinfeld M.C., Cromwell H.C., Kroes R.A., Moskal J.R. (2009). The effects of selective breeding for differential rates of 50-kHz ultrasonic vocalizations on emotional behavior in rats. Dev. Psychobiol..

[67] Burgdorf J., Panksepp J., Brudzynski S.M., Kroes R., Moskal J.R. (2005). Breeding for 50-kHz positive affective vocalization in rats. Behav. Genet..

[68] Webber E.S., Harmon K.M., Beckwith T.J., Peña S., Burgdorf J., Panksepp J., Cromwell H.C. (2012). Selective breeding for 50 kHz ultrasonic vocalization emission produces alterations in the ontogeny and regulation of rough-and-tumble play. Behav. Brain Res..

[69] Burgdorf J., Moskal J.R., Brudzynski S.M., Panksepp J. (2013). Rats selectively bred for low levels of play-induced 50 kHz vocalizations as a model for autism spectrum disorders: A role for NMDA receptors. Behav. Brain Res..

[70] Brunelli S.A., Nie R., Whipple C., Winiger V., Hofer M.A., Zimmerberg B. (2006). The effects of selective breeding for infant ultrasonic vocalizations on play behavior in juvenile rats. Physiol. Behav..

[71] Dichter G.S., Brunelli S.A., Hofer M.A. (1996). Elevated plus-maze behavior in adult offspring of selectively bred rats. Physiol. Behav..

[72] Brunelli S.A. (2005). Selective breeding for an infant phenotype: Rat pup ultrasonic vocalization (USV). Behav. Genet..

[73] Lukas M., Wöhr M. (2015). Endogenous vasopressin, innate anxiety, and the emission of pro-social 50-kHz ultrasonic vocalizations during social play behavior in juvenile rats. Psychoneuroendocrinology.

[74] Schneider P., Pätz M., Spanagel R., Schneider M. (2016). Adolescent social rejection alters pain processing in a CB1 receptor dependent manner. Eur. Neuropsychopharmacol..

[75] Burgdorf J., Kroes R.A., Weiss C., Oh M.M., Disterhoft J.F., Brudzynski S.M., Panksepp J., Moskal J.R. (2011). Positive emotional learning is regulated in the medial prefrontal cortex by GluN2B-containing NMDA receptors. Neuroscience.

[76] Moskal J.R., Burgdorf J., Kroes R.A., Brudzynski S.M., Panksepp J. (2011). A novel NMDA receptor glycine-site partial agonist, GLYX-13, has therapeutic potential for the treatment of autism. Neurosci. Biobehav. Rev..

[77] Burgdorf J., Kroes R.A., Beinfeld M.C., Panksepp J., Moskal J.R. (2010). Uncovering the molecular basis of positive affect using rough-and-tumble play in rats: A role for insulin-like growth factor I. Neuroscience.

[78] Burgdorf J., Panksepp J., Beinfeld M.C., Kroes R.A., Moskal J.R. (2006). Regional brain cholecystokinin changes as a function of rough-and-tumble play behavior in adolescent rats. Peptides.

[79] Willey A.R., Varlinskaya E.I., Spear L.P. (2009). Social interactions and 50 kHz ultrasonic vocalizations in adolescent and adult rats. Behav. Brain Res..

[80] Waddell J., Yang T., Ho E., Wellmann K.A., Mooney S.M. (2016). Prenatal ethanol exposure and whisker clipping disrupt ultrasonic vocalizations and play behavior in adolescent rats. Brain Sci..

[81] Wellmann K.A., George F., Brnouti F., Mooney S.M. (2015). Docosahexaenoic acid partially ameliorates deficits in social behavior and ultrasonic vocalizations caused by prenatal ethanol exposure. Behav. Brain Res..

[82] Wellmann K.A., Varlinskaya E.I., Mooney S.M. (2014). D-Cycloserine ameliorates social alterations that result from prenatal exposure to valproic acid. Brain Res. Bull..

[83] Raza S., Himmler B.T., Himmler S.M., Harker A., Kolb B., Pellis S.M., Gibb R. (2015). Effects of prenatal exposure to valproic acid on the development of juvenile-typical social play in rats. Behav. Pharmacol..

[84] Shahrier M.A., Wada H. (2020). Effects of prenatal ethanol exposure on acoustic characteristics of play fighting-induced ultrasonic vocalizations in juvenile rats. Neurotoxicology.

[85] Gzielo K., Potasiewicz A., Hołuj M., Litwa E., Popik P., Nikiforuk A. (2020). Valproic acid exposure impairs ultrasonic communication in infant, adolescent and adult rats. Eur. Neuropsychopharmacol..

[86] Gzielo K., Potasiewicz A., Litwa E., Piotrowska D., Popik P., Nikiforuk A. (2021). The effect of maternal immune activation on social play-induced ultrasonic vocalization in rats. Brain Sci..

[87] Kentner A.C., Scalia S., Shin J., Migliore M.M., Rondón-Ortiz A.N. (2018). Targeted sensory enrichment interventions protect against behavioral and neuroendocrine consequences of early life stress. Psychoneuroendocrinology.

[88] Wöhr M., Schwarting R.K.W. (2007). Ultrasonic communication in rats: Can playback of 50-kHz calls induce approach behavior?. PLoS ONE.

[89] Wöhr M., Schwarting R.K.W. (2008). Maternal care, isolation-induced infant ultrasonic calling, and their relations to adult anxiety-related behavior in the rat. Behav. Neurosci..

[90] Wöhr M., Schwarting R.K.W. (2012). Testing social acoustic memory in rats: Effects of stimulus configuration and long-term memory on the induction of social approach behavior by appetitive 50-kHz ultrasonic vocalizations. Neurobiol. Learn. Mem..

[91] Willadsen M., Seffer D., Schwarting R.K.W., Wöhr M. (2014). Rodent ultrasonic communication: Male prosocial 50-kHz ultrasonic vocalizations elicit social approach behavior in female rats (Rattus norvegicus). J. Comp. Psychol..

[92] Engelhardt K.A., Schwarting R.K.W., Wöhr M. (2018). Mapping trait-like socio-affective phenotypes in rats through 50-kHz ultrasonic vocalizations. Psychopharmacology.

[93] Seffer D., Rippberger H., Schwarting R.K.W., Wöhr M. (2015). Pro-Social 50-kHz ultrasonic communication in rats: Post-weaning but not post-adolescent social isolation leads to social impairments-phenotypic rescue by re-socialization. Front. Behav. Neurosci..

[94] Brenes J.C., Lackinger M., Höglinger G.U., Schratt G., Schwarting R.K.W., Wöhr M. (2016). Differential effects of social and physical environmental enrichment on brain plasticity, cognition, and ultrasonic communication in rats. J. Comp. Neurol..

[95] Berz A., de Souza C.P., Wöhr M., Schwarting R.K.W. (2021). Limited generalizability, pharmacological modulation, and state-dependency of habituation towards pro-social 50-kHz calls in rats. IScience.

[96] Sadananda M., Wöhr M., Schwarting R.K.W. (2008). Playback of 22-kHz and 50-kHz ultrasonic vocalizations induces differential c-fos expression in rat brain. Neurosci. Lett..

[97] Willuhn I., Tose A., Wanat M.J., Hart A.S., Hollon N.G., Phillips P.E., Schwarting R.K.W., Wöhr M. (2014). Phasic dopamine release in the nucleus accumbens in response to pro-social 50 kHz ultrasonic vocalizations in rats. J. Neurosci..

[98] Engelhardt K.A., Fuchs E., Schwarting R.K.W., Wöhr M. (2017). Effects of amphetamine on pro-social ultrasonic communication in juvenile rats: Implications for mania models. Eur. Neuropsychopharmacol..

[99] Wöhr M., Schwarting R.K.W. (2009). Ultrasonic communication in rats: Effects of morphine and naloxone on vocal and behavioral responses to playback of 50-kHz vocalizations. Pharmacol. Biochem. Behav..

[100] Berg E.L., Copping N.A., Rivera J.K., Pride M.C., Careaga M., Bauman M.D., Berman R.F., Lein P.J., Harony-Nicolas H., Buxbaum J.D. (2018). Developmental social communication deficits in the Shank3 rat model of Phelan-McDermid syndrome and autism spectrum disorder. Autism Res..

[101] Bourgeron T. (2015). From the genetic architecture to synaptic plasticity in autism spectrum disorder. Nat. Rev. Neurosci..

[102] Berg E.L., Pride M.C., Petkova S.P., Lee R.D., Copping N.A., Shen Y., Adhikari A., Fenton T.A., Pedersen L.R., Noakes L.S. (2020). Translational outcomes in a full gene deletion of ubiquitin protein ligase E3A rat model of Angelman syndrome. Transl. Psychiatry.

[103] Berg E.L., Ching T.M., Bruun D.A., Rivera J.K., Careaga M., Ellegood J., Lerch J.P., Wöhr M., Lein P.J., Silverman J.L. (2020). Translational outcomes relevant to neurodevelopmental disorders following early life exposure of rats to chlorpyrifos. J. Neurodev. Disord..

[104] Kircher T., Wöhr M., Nenadic I., Schwarting R.K.W., Schratt G., Alferink J., Culmsee C., Garn H., Hahn T., Müller-Myhsok B. (2019). Neurobiology of the major psychoses: A translational perspective on brain structure and function-the FOR2107 consortium. Eur. Arch. Psychiatry Clin. Neurosci..

[105] Trezza V., Damsteegt R., Vanderschuren L.J. (2009). Conditioned place preference induced by social play behavior: Parametrics, extinction, reinstatement and disruption by methylphenidate. Eur. Neuropsychopharmacol..

[106] Pellis S.M., Pellis V.C., McKenna M.M. (1994). Feminine dimension in the play fighting of rats (Rattus norvegicus) and its defeminization neonatally by androgens. J. Comp. Psychol..

[107] Albert D.J., Walsh M.L., Gorzalka B.B., Siemens Y., Louie H. (1986). Testosterone removal in rats results in a decrease in social aggression and a loss of social dominance. Physiol. Behav..

[108] Wöhr M., Borta A., Schwarting R.K.W. (2005). Overt behavior and ultrasonic vocalization in a fear conditioning paradigm: A dose-response study in the rat. Neurobiol. Learn. Mem..

[109] Sykes L., Haddon J., Lancaster T.M., Sykes A., Azzouni K., Ihssen N., Moon A.L., Lin T.E., Linden D.E., Owen M.J. (2019). Genetic variation in the psychiatric risk gene CACNA1C modulates reversal learning across species. Schizophr. Bull..

[110] Moon A.L., Brydges N.M., Wilkinson L.S., Hall J., Thomas K.L. (2020). Cacna1c hemizygosity results in aberrant fear conditioning to neutral stimuli. Schizophr. Bull..

[111] Moon A.L., Haan N., Wilkinson L.S., Thomas K.L., Hall J. (2018). CACNA1C: Association with psychiatric disorders, behavior, and neurogenesis. Schizophr. Bull..

[112] Tigaret C.M., Lin T.E., Morrell E.R., Sykes L., Moon A.L., O’Donovan M.C., Owen M.J., Wilkinson L.S., Jones M.W., Thomas K.L. (2021). Neurotrophin receptor activation rescues cognitive and synaptic abnormalities caused by hemizygosity of the psychiatric risk gene Cacna1c. Mol. Psychiatry.

[113] Łopuch S., Popik P. (2011). Cooperative behavior of laboratory rats (Rattus norvegicus) in an instrumental task. J. Comp. Psychol..

[114] Wöhr M. (2021). Measuring mania-like elevated mood through amphetamine-induced 50-kHz ultrasonic vocalizations in rats. Br. J. Pharmacol..

